# Assessment of Machine Learning Methods to Predict Massive Blood Transfusion in Trauma

**DOI:** 10.1007/s00268-023-07098-y

**Published:** 2023-06-30

**Authors:** Matt Strickland, Anthony Nguyen, Shinyi Wu, Sze-Chuan Suen, Yanda Mu, Juan Del Rio Cuervo, Brandon J. Shin, Tej Kalakuntla, Cameron Ghafil, Kazuhide Matsushima

**Affiliations:** 1 Department of Surgery, University of Southern California, LAC+USC Medical Center (The work was done at LAC+USC Medical Center), Los Angeles, CA USA; 2 Department of Surgery, University of Alberta, Edmonton, AB Canada; 3 Daniel J. Epstein Department of Industrial and Systems Engineering, University of Southern California, Los Angeles, CA USA; 4 Division of Acute Care Surgery, Department of Surgery, University of Southern California, 2051 Marengo Street, Inpatient Tower, C5L100, Los Angeles, CA 90033 USA

## Abstract

**Background:**

Accurately predicting which patients are most likely to benefit from massive transfusion protocol (MTP) activation may help patients while saving blood products and limiting cost. The purpose of this study is to explore the use of modern machine learning (ML) methods to develop and validate a model that can accurately predict the need for massive blood transfusion (MBT).

**Methods:**

The institutional trauma registry was used to identify all trauma team activation cases between June 2015 and August 2019. We used an ML framework to explore multiple ML methods including logistic regression with forward and backward selection, logistic regression with lasso and ridge regularization, support vector machines (SVM), decision tree, random forest, naive Bayes, XGBoost, AdaBoost, and neural networks. Each model was then assessed using sensitivity, specificity, positive predictive value, and negative predictive value. Model performance was compared to that of existing scores including the Assessment of Blood Consumption (ABC) and the Revised Assessment of Bleeding and Transfusion (RABT).

**Results:**

A total of 2438 patients were included in the study, with 4.9% receiving MBT. All models besides decision tree and SVM attained an area under the curve (AUC) of above 0.75 (range: 0.75–0.83). Most of the ML models have higher sensitivity (0.55–0.83) than the ABC and RABT score (0.36 and 0.55, respectively) while maintaining comparable specificity (0.75–0.81; ABC 0.80 and RABT 0.83).

**Conclusions:**

Our ML models performed better than existing scores. Implementing an ML model in mobile computing devices or electronic health record has the potential to improve the usability.

## Introduction

Over the last few decades, a massive transfusion protocol (MTP) has become a widespread tool in the management of severely injured patients to help ensure that blood products are coordinated and delivered in an expeditious manner while adhering to an optimal ratio of each component of transfusion therapy. There is good evidence that these protocols help clinicians provide earlier and more balanced resuscitation and their use has been associated with improved patient survival [1, 2]. However, MTP activations are resource-intensive events. Not only do they consume large amounts of blood products, but they may also require the allocation of specific human resources, such as blood bank technologists or porters, for hours. Thus, there is a special interest in accurately determining which patients are most likely to benefit from activation of MTP.

Several scoring systems have been created to help clinicians determine when MTP should be activated [3]. The ideal tool would balance sensitivity and specificity for the need for massive blood transfusion (MBT) so that all patients whose outcome depends on the timely activation of MTP would receive it, while excluding patients who would not benefit. Generally, these existing scores were derived from small- to medium-sized trauma patient cohorts. Inputs vary between scores, with some using only vital signs, some relying only on variables available at the bedside in the Emergency Department (ED), and others requiring laboratory values that would need some time process [4–7]. The calculation complexity also varies between these scores and this added burden may explain why the most cited score, the Assessment of Bleeding Consumption (ABC), is also one of the simplest [6].

Several factors have changed in the past decade which justifies another approach at predicting need for MBT. The number of trauma centers with MTPs has continued to increase [8] and even many small centers now have MTPs [9]. Clinicians in these smaller centers, where MTP is activated more rarely, are more likely to benefit from decision aids. In addition, the ubiquity of intelligent electronic health records (EHR) and stand-alone smartphone applications means that more complex tools may now be practical. Finally, in the last two decades, machine learning (ML) techniques have become increasingly sophisticated, accessible, and reported in medical applications. ML, broadly, uses a number of mathematical methods to process input data and create an output, commonly a prediction or classification. [10]. The purpose of this study was to investigate whether modern ML methods can be used to create a more accurate tool to predict the need for MTP. We hypothesized that ML models using only variables available in the period of the initial trauma assessment can predict the need for MBT more accurately compared to the currently used scoring systems.

## Methods

### Study design and patients

This is a retrospective study conducted at a high-volume, urban Level 1 Trauma Center. After approved by the Institutional Review Board, the institutional trauma registry was queried to identify eligible patients and retrieve the data between June 1, 2015 and August 31, 2019. Further information was collected from the EHR and digital picture archiving system. The study population included all patients (age ≥ 16 years) who presented as trauma team activations (TTA). The TTA criteria at our institution are included in Supplemental Table 1. Patients were excluded if they presented without any signs of life or if they had missing Glasgow Coma Scale (GCS) or ED vital sign values.

**Table 1:** Valuables included in the machine-learning model

Variable	Total patients (*n* = 2438)
Median age, year (IQR)	37 (26–56)
Penetrating mechanism (%)	955 (39.2)
Blunt mechanism (%)	1,511 (62.0)
Median SBP, mmHg (IQR)	135 (118–152)
Median DBP, mmHg (IQR)	90 (74–104)
Median respiratory rate (IQR)	20 (16–23)
Median oxygen saturation, % (IQR)	100 (97–100)
Median GCS eye opening (IQR)	4 (4–4)
Median GCS motor response (IQR)	6 (5–6)
Median GCS verbal response (IQR)	5 (5–3)
Positive FAST (%)	383 (15.7)
Positive pelvis X-ray (%)	287 (11.7)

IQR: interquartile range, SBP: systolic blood pressure, DBP: diastolic blood pressure,

GCS: Glasgow Coma Scale, FAST: focused assessment with sonography for trauma

### Data collection

Variables available during the initial trauma assessment in the ED were collected (age, sex, body mass index [BMI], mechanism of injury, pre-hospital and ED vital signs, and GCS). Results of the extended focused assessment with sonography for trauma (eFAST) were extracted from the EHR including the location of any positive results (thorax, pericardium, abdomen). Portable pelvis x-ray was reviewed by board-certified surgeons (MS, KM) and assessed for presence of visible pelvic fractures. In the primary analysis, pelvis x-ray was not used in the models because it was not routinely performed. In a sensitivity analysis, pelvis x-ray results were included to evaluate for changes in prediction model performances. Finally, blood transfusion data were obtained including number of units and timing of transfusions, type of product transfused, and whether MTP was activated.

### Statistical analysis

The outcome of interest was need for MBT which was defined as the need for ≥10 units of packed red blood cells (PRBC) in the first 24 h after arrival. We tested commonly used ML techniques including regression (simple and penalized) and decision trees (single tree and random forest) which are the generally interpretable models. We expanded our assessment of ML algorithms by implementing support vector machines (SVMs), naïve Bayes, boosting techniques such as XGBoost and AdaBoost, and neural networks.

All models were validated using a fivefold cross validation process. Under a fivefold cross validation, the data were split into five partitions. The full analysis was then run five times where during each run, a different partition was used as the testing data while the remaining partitions were used as the training data. A model that performs well should show similar performance results across each of the five runs. Some models required a threshold value for classification (e.g., logistic regression). In those cases, we present each model’s performance when we selected a threshold value that minimizes the distance from the receiver operating characteristics (ROC) curve to perfect sensitivity and specificity. In practice, different thresholds can be selected to reflect the local preferences for higher sensitivity or specificity. We evaluated model performance by looking at the following metrics of interest across our cross validation: area under the curve (AUC), sensitivity, specificity, positive predicted value (PPV) and negative predicted value (NPV). In addition to comparing our prediction models to each other, we also compared our models’ performance to the ABC score [6] and the Revised Assessment of Bleeding and Transfusion (RABT) score [7], when applied to our patient set.

## Results

**Fig. 1:**
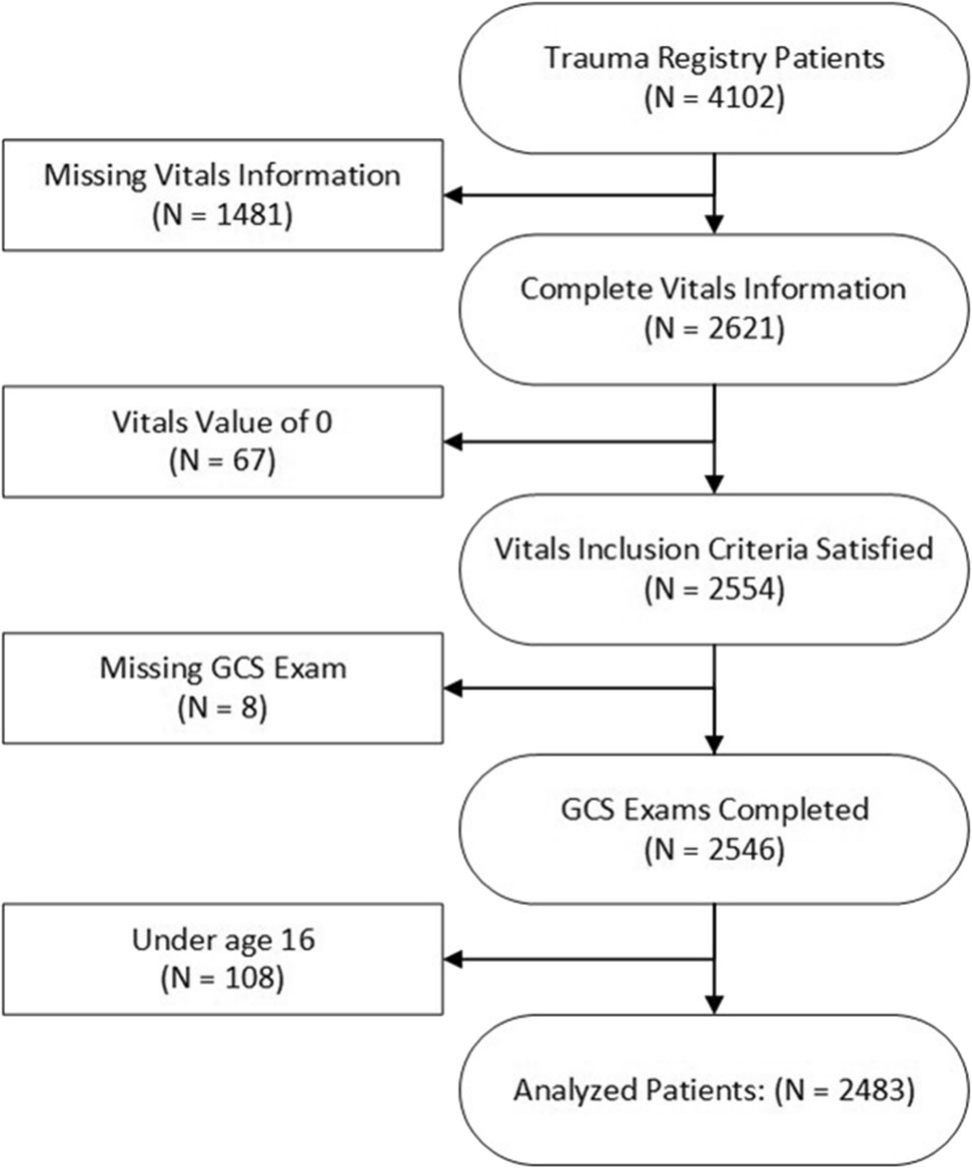
Patient flow diagram. GCS: Glasgow Coma Scale

During the study period, 4102 TTA patients were identified in our trauma registry. After excluding patients under age 16 years, with no signs of life upon arrival, and with missing information, a total of 2,483 patients were included for analysis (Fig. 1). The median age was 37 years, median SBP of 135 mmHg, and DBP of 90 mmHg (Table 1). The mean injury severity score was 13. Approximately 98% of the patients had an eFAST examination performed with 15.7% of those results being positive. Only 37% of the patients underwent a pelvis x-ray, and after verifying against patient charts, 11.7% of those patients had positive results. While MTP was activated 233 times (9.4%), only 121 (4.9%) required MBT within the first 24 h of arrival. Full descriptive statistics are shown in Table 2.

**Table 2:** Patient outcomes

Outcomes	Total patients (n = 2438)
In-hospital mortality (%)	9.9%
Massive blood transfusion* (%)	5.0%
MTP activated (%)	9.4%
Median PRBC within 4 h, mL (IQR)	0 (0–291.5)
Median PRBC within 24 h, mL (IQR)	0 (0–543.8)
Median plasma within 4 h, mL (IQR)	0 (0–0)
Median plasma within 24 h, mL (IQR)	0 (0–0)
Median platelets within 4 h, mL (IQR)	0 (0–0)
Median platelets within 24 h, mL (IQR)	0 (0–0)

^*^ > 10 units of PRBC within 24 h after arrival

MTP: massive transfusion protocol, PRBC: packed red blood cell, IQR: interquartile range

**Fig. 2:**
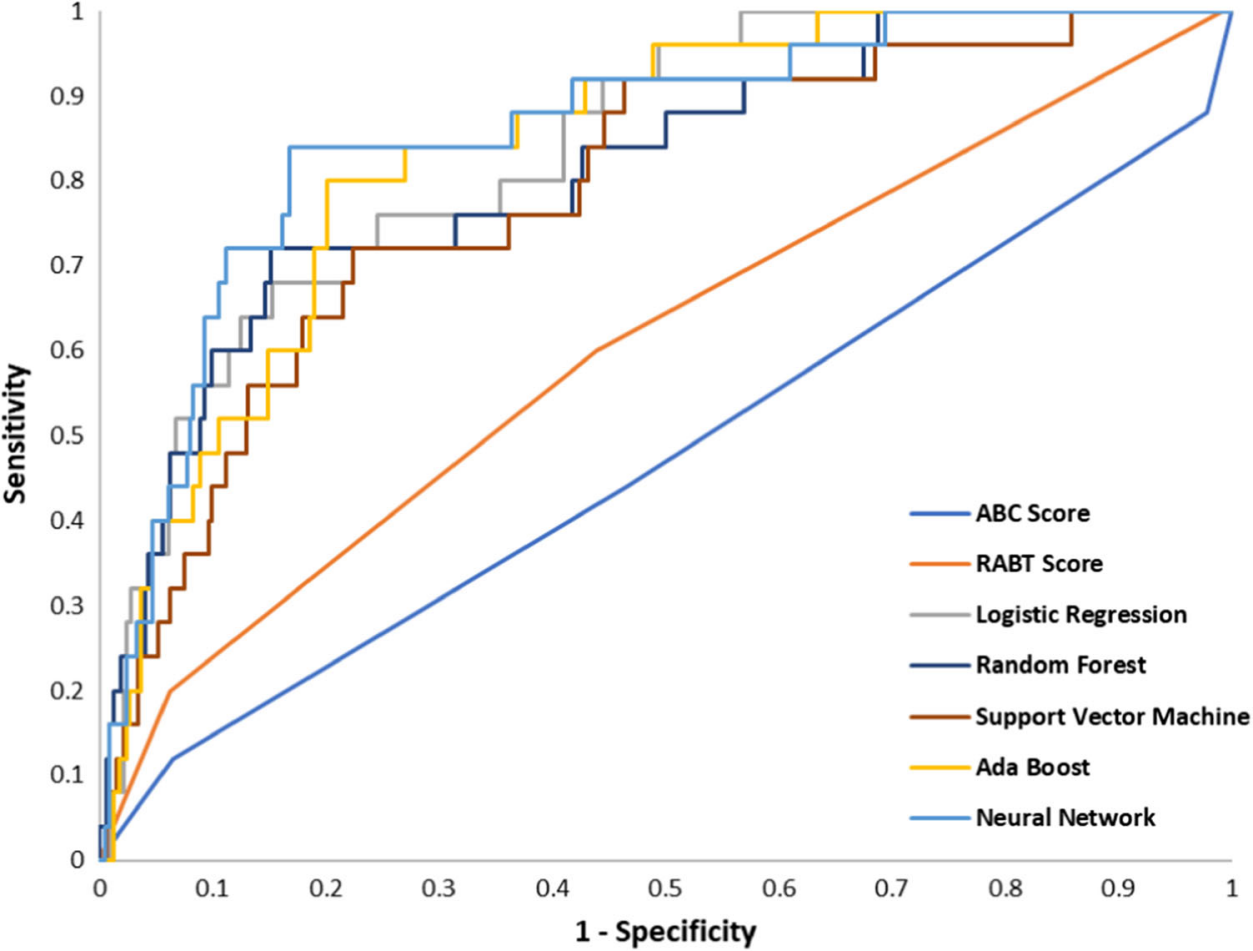
Comparison of ML models using ROC curves. ML: machine learning, ROC: receiver operating characteristics, ABC: Assessment of Blood Consumption, RABT: Revised Assessment of Bleeding and Transfusion

In our study population, we observed that the ABC score had a sensitivity of 0.36, specificity of 0.80, PPV of 0.08, and NPV of 0.96. The RABT score had values of 0.55, 0.83, 0.14, and 0.23 for sensitivity, specificity, PPV, and NPV, respectively. Compared with these scores, all ML models had comparable or higher sensitivity. All models, except for SVM. Naïve Bayes, and Neural networks had comparable specificity, PPV, and NPV. Full performance metrics with mean and standard errors from the cross-validation results are shown in Table 3. The ROC curves for all tested models are shown in Fig. 2. In a sensitivity analysis where all ML models are re-run using the pelvis X-ray information, we find that our model performance generally improves with logistic regression reaming as a high performing model. Model performance metrics for the sensitivity analysis are presented in the supplementary document in Supplemental Table 2.

**Table 3:** Model performance for predicting need for massive transfusion protocol

Model	AUC (SE)	Sensitivity (SE)	Specificity (SE)	PPV (SE)	NPV (SE)
ABC score	0.52 (0.028)	0.51 (0.061)	0.52 (0.024)	0.05 (0.004)	0.95 (0.005)
RABT score	0.64 (0.051)	0.67 (0.094)	0.52 (0.035)	0.07 (0.007)	0.97 (0.008)
Logistic regression	**0.83 (0.034)**	**0.81 (0.033)**	0.75 (0.019)	0.15 (0.012)	**0.99 (0.003)**
Lasso	0.77 (0.067)	0.72 (0.074)	0.75 (0.041)	0.13 (0.023)	0.98 (0.005)
Ridge	0.77 (0.067)	0.73 (0.077)	0.74 (0.023)	0.13 (0.012)	0.98 (0.005)
CART	0.69 (0.037)	0.48 (0.068)	**0.89 (0.014)**	0.19 (0.034)	0.97 (0.003)
RF	0.81 (0.039)	0.77 (0.033)	0.76 (0.102)	0.16 (0.056)	0.98 (0.003)
SVM	0.66 (0.011)	0.6 (0.035)	0.7 (0.056)	0.1 (0.023)	0.97 (0.004)
XGBoost	0.8 (0.035)	0.74 (0.079)	0.79 (0.066)	**0.16 (0.027)**	0.98 (0.004)
Naive Bayes	0.77 (0.048)	0.74 (0.036)	0.71 (0.047)	0.12 (0.019)	0.98 (0.004)
AdaBoost	0.79 (0.059)	0.77 (0.076)	0.75 (0.061)	0.14 (0.024)	0.98 (0.004)
Neural network	0.81 (0.053)	0.79 (0.062)	0.77 (0.045)	0.15 (0.034)	0.99 (0.004)

^*^Bolded numbers represent the highest value seen across all models

ABC: Assessment of Blood Consumption, RABT: Revised Assessment of Bleeding and Transfusion, CART: Classification and Regression Tree, RF: Radio Frequency, SVM: Support Vector Machine, AUC: Area Under the Curve, PPV: positive predictive value, NPV: negative predictive value, SE: Standard Error

## Discussion

The current study showed that most ML methods outperform the ABC score and RABT score in predicting the need for MBT in TTA patients. We used a large patient dataset, approximately five times larger than those used by other groups to develop scoring systems and limited our inclusion to objective data available early in the initial trauma assessment. A machine learning framework was then used to evaluate numerous ML tools and compare them in terms of test characteristics. Although one other study has used a modern ML method [11], we report the first multi-method approach, searching to optimize prediction over a broad range of techniques.

Predicting who will require MBT has been an area of ongoing research for nearly two decades. Early activation of MTP is associated with improved mortality in several studies [1, 2] and, as these protocols have become widespread, the need for decision aids has presumably increased. At least 15 scoring systems predicting MBT requirement have been described and the pros and cons of each of these have also been reviewed [12, 13]. Notably, most of these scoring systems are derived retrospectively from single-center experiences. Prior work has demonstrated that, in general, the more variables are considered, the better the score performs. The simplest scores use only physiologic data or a combination of physiologic data and information about mechanism [14, 15]. More comprehensive scores use data obtained from the FAST exam, laboratory results, and plain radiography [5–7, 16]. While additional variables tend to improve score performance in terms of predictive power, waiting for laboratory studies to result or medical imaging may lead to delays in MTP activation which ultimately could erode the benefit of early activation. In this study, we therefore focused on patient characteristics that are readily available early upon arrival to the trauma center, to preclude delay for laboratory results. The number of variables, while more comprehensive than the simplest, most popular scores currently in use, was also selected to not be too large so as to become onerous to use in a trauma setting with the help of an electronic app.

The majority of existing scoring systems were derived by using regression methods and relatively small populations. The ABC score, for example, was derived from a cohort of 596 patients with 77 massive transfusion events [6]. The RABT score used a population of 380 patients and 102 massive transfusions [7]. Many of the existing scores dichotomize the input variables to make calculation simpler, at the expense of accuracy. As an example, the ABC score assigns one point for SBP ≤ 90 mmHg which means that, all other factors being equal, a patient with SBP of 91 mmHg is regarded the same way as a patient with an SBP of 120 mmHg. This desire for simplicity is pervasive among risk scores. This was a necessary feature of classic scores such as Ranson’s criteria for acute pancreatitis mortality or the Child–Pugh score for cirrhosis mortality where clinicians simply did not have access to computing power to perform more sophisticated calculations [17, 18]. With the widespread availability of smartphones, tables, and other mobile devices in the clinical setting and the increased adoption of EHR systems, modern scoring systems may benefit from more complex models. Our group has been working on the development of a mobile app to be used by clinicians in real time without increasing their workload. Given the paucity of data on the use of mobile apps in a highly stressful medical environment, future studies should evaluate usability of the prediction app in the acute trauma setting.

In contrast to existing scoring systems, the current study explored different ML techniques for MBT prediction. ML has brought standardized techniques by which to evaluate and compare statistical models beyond those which were traditionally used. AUC, sensitivity, specificity, PPV, and NPV are widely used and understood metrics by which multiple models can be transparently compared. It has also increased the acceptance of cross validation and other methods to identify over- and underfitting, which may be particularly valuable when data comes from a single center and may not be generalizable a priori. In this work, we examine the performance of existing methods (such as ABC score, etc.) against ML methods on these metrics using fivefold cross validation to provide multiple perspectives in understanding how this data might perform in a real-world setting.

Our results identified 3 ML models that were comparable or outperformed the well-accepted ABC score and RABT score across all metrics: logistic regression, Random Forest, and AdaBoost. The remaining models still perform better in terms of sensitivity but have noticeably lower specificity. Given that many of these models performed very similarly, choosing the optimal model to build into production depends in part on human factors and the costs of over- or under-classifying. Ultimately, the final model we would recommend is the complete simple logistic regression without applying any variable selection or regularization. We identify this as the most preferable model for three reasons: (1) The complete simple logistic regression performance is as good as, if not better, than the other ML approaches tested, (2) A simple logistic regression is highly interpretable and more familiar to clinicians, which will assist in future efforts toward implementation. For example, our regression results confirm that a higher heart rate is an indicator of increased need for MBT, (3) Removing variables that a physician may deem to be significant can result in skepticism regarding the model, and translate to further challenges in implementation. Additionally, as previously discussed, we left out pelvic x-ray results because it is not consistently performed in our study cohort, thus rendering the model useless if pelvic results were required. Sensitivity analysis did however show that incorporating pelvic x-ray results can improve predictive performance.

Our study has several limitations. First, our training and validation datasets were composed of retrospective data collected at discrete points in time. Although this remains how data are stored in trauma registries, clearly some information was lost through this simplification. Having multiple sets of physiologic data or even continuous information, for example, might lead to better prediction as has been demonstrated in other ML applications [19]. Second, we included all patient data accessible retrospectively, but other streams available to the clinician at the point of care were not included. These include subtleties about the mechanism, patient medications, and the patient’s general appearance which, especially to expert clinicians, may carry strong predictive value. Third, we had to handle some variables cautiously because of associated information that would erode at the generalizability of our results. At our center, for example, pelvic x-ray is not routinely performed because our time to computed tomography (CT) is sufficiently low that we consider it unnecessary in most patients. Thus, plain film is often reserved for patients who are particularly unwell and who, in the trauma attending’s opinion, may not be stable enough for CT. In early iterations of our algorithms, this resulted in a strong positive association between merely receiving a pelvic X-ray and need for massive transfusion. The result is that we had to exclude an important potential source of data because of this confounding effect. Finally, like most studies attempting to create a prediction tool, there is the risk of overtraining to our data set and not being able to reproduce our results on another population of patients.

## Conclusions

Our results suggest that the use of modern ML methods can significantly improve the accuracy in predicting the need for MBT. However, this improvement must be validated and the feasibility of implementing these algorithms in the trauma bay environment must be explored in future studies.
